# Effect of Correlations in Swarms on Collective Response

**DOI:** 10.1038/s41598-017-09830-w

**Published:** 2017-09-04

**Authors:** David Mateo, Yoke Kong Kuan, Roland Bouffanais

**Affiliations:** Singapore University of Technology and Design, 8 Somapah Road, Singapore, 487372 Singapore

## Abstract

Social interaction increases significantly the performance of a wide range of cooperative systems. However, evidence that natural swarms limit the number of interactions suggests potentially detrimental consequences of excessive interaction. Using a canonical model of collective motion, we find that the collective response to a dynamic localized perturbation–emulating a predator attack–is hindered when the number of interacting neighbors exceeds a certain threshold. Specifically, the effectiveness in avoiding the predator is enhanced by large integrated correlations, which are known to peak at a given level of interagent interaction. From the network-theoretic perspective, we uncover the same interplay between number of connections and effectiveness in group-level response for two distinct decision-making models of distributed consensus operating over a range of static networks. The effect of the number of connections on the collective response critically depends on the dynamics of the perturbation. While adding more connections improves the response to slow perturbations, the opposite is true for fast ones. These results have far-reaching implications for the design of artificial swarms or interaction networks.

## Introduction

Social interaction is critical for swarms to perform an effective and coordinated response to changing environments. Social activity and the associated transmission of information through the interaction network have recently attracted considerable attention in a wide range of complex systems: from the biological realm–flock of birds^1, 2^, school of fish^3–6^, swarm of insects^7–9^, and human crowds^10^–to social networks^11^, and artificial multi-agent systems such as the power grid^12, 13^ or robotic swarms^14, 15^. The characteristics of the interaction network are known to strongly affect the swarm dynamics^16–18^ and, in particular, its capacity to respond to local perturbations^4, 6, 19, 20^.

Increasing the number of interactions between agents usually improves the performance of the collective, but it is known that most natural swarms operate with a limited number of connections. For instance, flocking starlings interact on average with 6 to 7 conspecifics^21^ and swarms of midges^8^ regulate their nearest-neighbor distance depending on the size of the swarm. Gordon *et al*.^22^ have shown that one species of ants (*L*. *fuliginosus*) regulate its rate of social encounters following: *(i)* changes in the nestmate density for undisturbed ant colonies, and *(ii)* the introduction of an external perturbation–workers from another colony–in the colony. This limited interaction appears to be a behavioral feature and not a direct result of physical limitations of their sensing capabilities. These findings suggest that natural swarms may tune the amount of interaction or number of connections in order to increase their capacity to collectively respond to environmental changes.

Classic phenomenological models of collective motion feature a critical point at a certain number of connections that maximizes the integrated correlation in the swarm^23^. The observed collective dynamics of midges^8^ provides experimental evidence that this swarm tunes the amount of interaction–inferred from density–in a way that maximizes correlations. This critical behavior may help explain why different social organisms seem to self-limit the number of connections, assuming that large integrated correlations do enhance the collective response for the benefit of biological functions such as predator avoidance or foraging.

From the theoretical standpoint, some models of decision-making dynamics predict that over-reliance on social information can render a collective unresponsive to changing circumstances^24, 25^. Models of consensus in mobile communicating agents have also shown that consensus can be reached more efficiently with a limited interaction range^26^.

Several empirical studies^19, 27, 28^ have shown that for social behaviors such as the adoption of new ideas or innovations to spread successfully over a network, agents must be exposed to multiple reinforcements coming from a minimum fraction of their connections. This behavioral spreading process is called *complex contagion*, in contrast with simple contagion processes such as the spread of information or diseases. It is now well known that these complex contagion processes are very sensitive to the topology of the network connecting agents. In particular, studies of complex contagion modeling the adoption of new behaviors^29, 30^ have shown that, in some contexts, adding connections decreases the spread of adoption.

Understanding the consequences of excessive connections is critical for achieving new functional predictions on collective animal behavior^3, 4^, and for the study of spreading of behaviors in networked systems such as online communities. From a technological viewpoint, developing a predictive theoretical framework to understand under which circumstances these effects appear is of paramount importance for the emerging field of large-scale swarm robotics^14, 15^.

Here, we investigate the relationship between the number of interacting agents and the responsiveness of the collective. First, we present an analysis of response to a predator attack using a classical model of collective motion in which self-propelled particles (SPPs) move by adjusting their direction of travel to that of their neighbors. We find that the capacity of the agents to avoid the predator is directly related to the integrated correlation of the collective, and thus peaks at a finite number of connections. Second, we extend the study beyond swarm dynamics by considering a general distributed decision-making model. We measure how the properties of the interaction network affect the dynamics of the collective decision when facing external influences. Finally, we develop an analytical framework based on linear time-invariant (LTI) theory that allows us to establish how the connectivity of a networked multi-agent system affects its overall responsiveness. This framework may be used to determine policies on interaction regulation for optimal dynamical response to a given perturbation.

## Results

### Collective response to a predator attack

**Figure 1:**
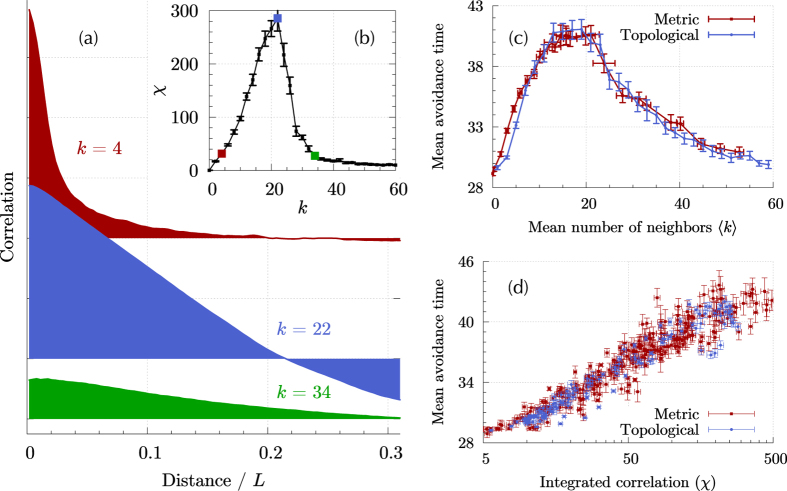
Interplay between correlations, number of connections, and predator avoidance. (**a**): Correlation in velocity fluctuations (Eq. (6)) for *N* = 2,048 topologically-interacting SPPs with number of neighbors or outdegree *k* = 4,22 and 34. The distance is measured in units of the computation box length *L*. (**b**): Integrated correlation (Eq. (7)) as a function of the outdegree *k*. (**c**): Mean number of iterations between two consecutive predator catches (avoidance time) as a function of the average number of neighbors 〈*k*〉 for metric (red, □) and topological (blue, ○) interactions. (**d**): Mean avoidance time as a function of *χ* for the equivalent unperturbed (in the absence of a predator) swarm.

A natural starting point to characterize the responsiveness of the collective motion is to study the connected correlation in fluctuations of the velocity^9^. We follow the framework developed by Attanasi *et al*.^7, 8^ and compute the connected correlation in velocity fluctuations *C*(*r*) (Eq. (6)) for a swarm composed of *N* = 2,048 SPPs while varying the number of neighbors *k*. In order to investigate the behavior of swarms displaying a high degree of alignment, we perform the calculations in the low noise regime (see Methods). The integrated correlation *χ* (Eq. (7)) is a measure of the total amount of correlation present in the system^7^. In the collective behavior literature, this quantity is commonly referred to as *susceptibility* in reference to its analog in equilibrium statistical physics, where the dissipation-fluctuation theorem establishes that the response of the system is proportional to this quantity. We find that a collective of SPPs following the Vicsek model in the ordered phase can exhibit a large integrated correlation if the number of neighbors *k* is set to an appropriate level (Fig. 1(b)).

The integrated correlation depends both on the span of correlations (how far in space the behavior of one agent influences another) and the intensity of correlations (how strong this influence is)^7^, and there is an intrinsic trade-off between them: an increase in the number of neighbors allows the information to travel farther through the network–increased correlation length–but causes each agent to be exposed to more information, thus decreasing the relevance of each individual signal–decreased correlation strength.

The correlation function is shown in Fig. 1(a) for three different values of the number of neighbors *k*, illustrating this trade-off. For small values of *k* (e.g. *k* = 4 in Fig. 1(a)), correlations are large but confined to short distances. As *k* increases, so does the spread of correlations and thus *χ*. Above a certain optimal number of neighbors, which is approximately ${k}^{\ast }\simeq 20$ for the particular configuration used in our calculation, the increase in spatial spread cannot compensate the reduction in correlation strength and *χ* diminishes with increasing number of neighbors.

**Figure 2:**
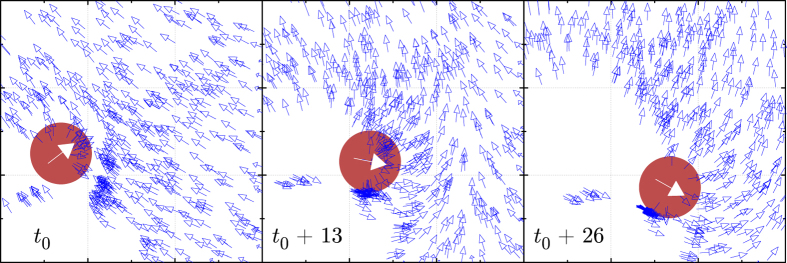
Collective evasive action induced by a predator attack. The SPPs (empty blue arrows) can only detect the threat (solid white arrow) inside the danger-detection region shown in red. In each consecutive frame, more agents outside this circle are able to flee without detecting the predator thanks to cooperative social behavior. Each square in the background grid has a side of length 10% that of the total computation box.

In order to illustrate how a highly aligned swarm benefits from a large *χ*, we have performed a model simulation of a predator attacking a group of SPPs following the Vicsek consensus and measured the survival rate of agents with different number of connections (see Methods). The emergent collective avoidance maneuver is shown in Fig. 2 for three selected snapshots of a predator attack and in movies M1–M3 (see Supplementary Note I). At *t*_0_ (leftmost frame), the predator starts the attack on a highly-aligned section of the swarm. Only the agents that detect the predator–those inside the red disk–react according to Eq. (8). After 13 iterations, agents outside the detection area are collectively reacting to the threat thanks to the social information transmitted through the swarm. After 26 iterations from the start of the attack, all agents in the vicinity of the predator perform a global evasive maneuver. Notice that the information transfer has taken place strikingly fast, which is in good agreement with recent empirical observations of collective turns in flocks of starlings^1^ and startled schools of fish^5, 31^.

It is worth pointing out that animals avoid predators using behaviors and strategies considerably more sophisticated than the model presented here. For instance, Rosenthal *et al*.^31^ have shown how schooling golden shiner fish use visual cues such as a fast change in speed to signal to other members the necessity to flee. However, this idealized model illustrates how classical concepts from statistical mechanics such as the connected correlation can be linked to aspects of a collective’s behavior crucial for the survival of its members. A similar case could be made for the capacity of swarms to forage for food, achieve optimal pattern formation, or other examples of animal collective behavior where a timely response to perturbations is critical^32, 33^.

The characteristic avoidance time for the swarm, defined as the average time elapsed between two consecutive catches by the predator, is shown in Fig. 1(c) as a function of the mean number of neighbors for both metric and topological interactions. In the latter case, the mean is exactly the imposed outdegree value *k*, while in the former the average is computed over all agents and iterations. Interestingly, both interactions yield essentially the same outcome. Starting from a noninteracting collective (〈*k*〉 = 0), the avoidance time grows with the number of neighbors up to a maximum value about 40% larger than the noninteracting time. From that optimal point at approximately 20 neighbors, the avoidance time monotonously decreases with increasing *k*, down to the value obtained for a noninteracting collective. The variations in the mean avoidance time are not due to variations in the mode but to the appearance of a heavy tail in the distribution of times (see Supplementary Note II). In order to better illustrate the effect of the number of connections in predator avoidance, Movies M1 to M3 in the Supplemental Information present the movement of the swarm for each of the three characteristic regimes: optimal *k* = 16, insufficient *k* = 4, and excessive *k* = 40 respectively.

Figure 1(d) shows a clear and systematic improvement of the swarm’s collective predator avoidance with increased *χ*. Taking the avoidance time as a measure of the capacity of a swarm to respond to localized perturbations, these simulations show that large correlations can translate into enhanced group-level responses beneficial to the swarm.

### Influence of connectivity on collective decision-making

The SPP model considered here is inarguably one of the simplest models of collective motion where the agents choose their heading in order to achieve a consensus in a distributed fashion. Despite its simplicity, the model features a rich phenomenology–from its critical behavior to its coupling of the dynamics *of* the interaction network with the dynamics *on* the network–that has been exhaustively documented in the literature. In order to abstract the effects of the collective decision-making process from the dynamics of the agents and the statistical description of the swarm, we turn our attention to a minimalist description of decision-making dynamics involving a set of fixed agents interacting through a static network and performing a distributed consensus protocol.

An archetypical minimalist model of decision-making is the so-called linear threshold model^34, 35^, which is a generalization of the simple majority vote model^36^. Using this model with different degrees of modularity, Nematzadeh *et al*.^37^ revealed that the network structure has a strong influence on the diffusion process. A similar conclusion was obtained by Centola^19^ using experiments on a specifically designed social network. In both cases, the effectiveness in diffusion was characterized by the influence of perturbations onto the asymptotic global state.

Here, we use the same model as Nematzadeh *et al*.^37^ to study the responsiveness of a decision-making process to perturbations. We characterize this response capacity using the polarization speed *c*, which is essentially the rate at which agents adapt their individual state to an induced perturbation detected only by a minority of informed agents (see Methods). These informed agents can be considered as “leaders” that drive the system from *P* = 0 to *P* = 1, much like the SPPs detecting the predator lead the swarm to perform a collective evasive maneuver or initiators can drive sheep herds to specific targets^38^.

**Figure 3:**
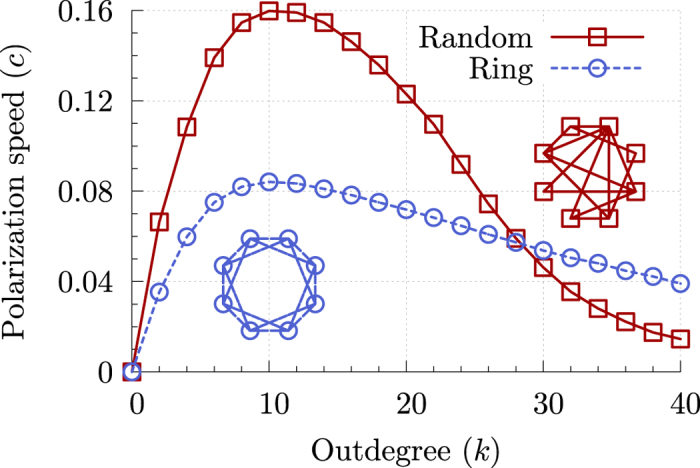
Polarization speed *c* for a linear threshold consensus protocol with threshold *θ* = 1/2 for a directed random network with fixed outdegree *k* (red, □), and an undirected one-dimensional regular lattice with *k*-nearest neighbors connectivity (blue, ○). The dynamics is triggered by switching 36% of the *N* = 2,048 agents to the state *s* = 1.

The polarization speed is shown in Fig. 3 for two extreme kinds of network wiring: a fixed-outdegree random directed network where each agent is randomly connected with *k* agents, and an undirected regular one-dimensional lattice (a ring) where each agent is connected with its *k* nearest neighbors. With both wirings, the polarization speed *c* is maximum for a finite outdegree *k* ^*^ which, for large systems, is fairly independent of the total number of agents *N* (see Supplementary Note III).

We have chosen a large enough number of informed leaders to guarantee that the system eventually reaches *P* = 1, i.e. a full polarization, in a finite time. This way, the polarization speed is determined by the short-time response capacity of the system, and not its asymptotic polarization at long times (for different ratios of informed leaders, see Supplementary Note IV). How fast the system reaches full polarization depends on the degree of the network and, as seen for the SPPs, too many connections hinder the performance of the system.

The results in Fig. 3 also reveal that the structure of the network can enhance or diminish the effects of connectivity on the response capacity. While the optimal outdegree *k* ^*^ = 10 is the same for completely random and highly structured networks, the polarization speed in the random network shows a larger sensitivity to the amount of connections. These results do not exhaustively prove the existence of such an optimal outdegree for any arbitrary topology. Nonetheless, it is reasonable to assume that most realistic examples of complex networked systems possess a network structure somewhere in between the two extreme cases considered here^39^ (see Supplementary Note V). A systematic study of the polarization speed for a wider collection of complex networks may reveal how the short-time response of a system is related to other properties of the interaction network such as degree distribution, average shortest connecting path, and clustering coefficient^40^.

### Responsiveness of cooperative multi-agent systems

In the previous sections, we have stressed the importance of distributed consensus problems in both biological and social systems. Both the SPP and the majority vote models provide excellent phenomenological frameworks to study how the level of connectivity among agents affects the responsiveness of cooperative systems. However, their phenomenological nature limits our ability to identify and characterize the underlying mechanisms responsible for the impaired collective responses under excessive connectivity.

Consensus and cooperation in networked multi-agent systems is a topic that is starting to receive significant attention in control theory and distributed computing owing to numerous possible engineering applications^41^. For instance, the power grid, urban traffic, arrays of distributed sensors, multi-robot systems, and social networks are some examples of collective systems requiring an effective response to local perturbations. The design of such systems–especially in the emerging field of swarm robotics–can be optimized using a theoretical framework that highlights the underlying mechanism and predicts under which conditions the detrimental effect of excessive connectivity will manifest. The LTI system theory provides one of the most elementary candidates for such a framework.

We consider a set of *N* + 1 agents performing a linear consensus protocol, and model the effects of local perturbations by setting one agent as a “leader” that does not participate to the local consensus dynamics albeit influencing agents connected to it. The leader-follower distributed linear consensus protocol presented in Eq. (11) is fairly standard^16–18, 41–43^, and can be used to analyze the capacity of the system to follow and adapt to fast changes in the behavior of the leading agent (see Methods). To simplify the problem as much as possible, static and undirected regular one-dimensional lattice topologies–*k* nearest neighbors with a ring topology–are considered for the network of interaction between agents.

Significant attention has been dedicated to the problem of convergence to consensus^18^ and controllability of multi-agent dynamics^44^ in the presence of complex network topologies–possibly switching–with directed or undirected information flow^41^. Here, given the simple topology of the static network, both convergence to consensus and controllability are guaranteed. Instead, our focus lies with the overall responsiveness of the collective in adapting to fast changes in the dynamics of the single leader–in control-theoretic terms, the input.

**Figure 4:**
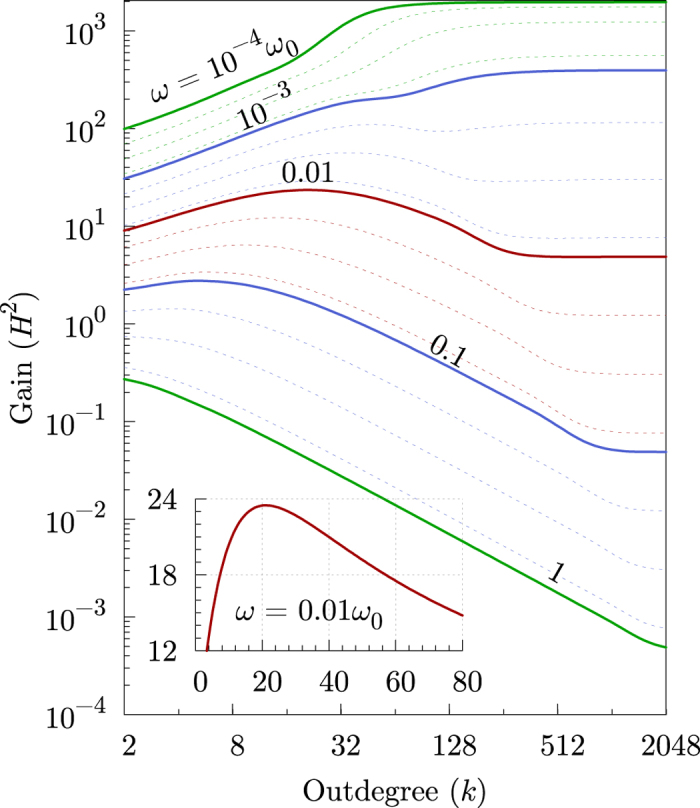
Responsiveness of a distributed consensus leader-follower protocol. The total amplitude gain for a system of *N* = 2,048 agents following a single leader as a function of the outdegree *k* (number of connections) is shown for several values of the leader’s oscillating frequency *ω*. Each solid line corresponds to a frequency equal to the agents’ natural frequency *ω*_0_ times the factor specified on top of the line. The dotted lines correspond to frequencies 2, 4, and 8 times larger than the one in the solid line above them. Inset: detail view of the gain for *ω* = 0.01*ω*_0_ in the region where the gain presents a maximum with respect to the outdegree.

To characterize the effects of varying levels of connectivity on the far-from-consensus responsiveness of the collective, Fig. 4 shows the response capacity of this system to oscillations of the leading agent as a function of the number of connections *k*, and for input oscillation frequencies *ω* spanning four orders of magnitude. This response capacity, measured by the total amplitude gain (see Methods), can be roughly interpreted as the number of agents that are capable of following the perturbation induced by the leader.

For such a rudimentary linear system, the response capacity exhibits a surprisingly rich structure. At low frequencies $\omega \ll {\omega }_{0}$, more connections always translates into an improvement in the system’s capacity to respond to perturbations. At high frequencies $\omega \gtrsim {\omega }_{0}$, the opposite is true: adding connections yields a systematic reduction in the system’s performance. A very interesting intermediate frequency regime is also observed (e.g. $\omega  \sim 0.01{\omega }_{0}$ in Fig. 4), where the responsiveness features a peak at a finite number of connections. The inset highlights that, in this intermediate frequency regime, the system can essentially double the amount of agents capable of following the leader by tuning the outdegree to its optimal value. This trend is reminiscent of what we have uncovered for the dependency of the correlations (Fig. 1(b)), mean avoidance time (Fig. 1(c)), and polarization speed (Fig. 3) with *k* in the previous phenomenological models.

Using the analytical expression for the gain, we can obtain general predictions for the responsiveness on arbitrary networks in the limits of low and high frequencies. For instance, in the limit of low frequencies, any system with an undirected interaction network (for directed ones, see Supplementary Note VI) will respond to perturbations of frequency $\omega \ll {\omega }_{0}$ as

1 $${H}_{\omega \ll {\omega }_{0}}^{2}={\Vert {{\bf{H}}}_{0}\Vert }^{2}-{\omega }^{2}{{\bf{H}}}_{0}^{\dagger }{{\bf{W}}}^{-2}{{\bf{H}}}_{0}+O({\omega }^{4}),$$

where **H**_0_ = **H**(0) = **W** ^−1^ **W**_*l*_ is the gain at *ω* = 0, **W** is related to the inter-agent connectivity, and **W**_*l*_ to the connectivity of the agents to the leader (see Methods). For any connected network, **H**_0_ is a vector with all components equal to 1^45^, and thus Eq. (1) can be written as

2 $${H}_{\omega \ll {\omega }_{0}}^{2}=N-{\omega }^{2}\sum _{i,j}{({{\bf{W}}}^{-2})}_{ij}+O({\omega }^{4}\mathrm{)}.$$

At high frequency, the gain is

3 $${H}_{\omega \gg {\omega }_{0}}^{2}={\omega }^{-2}{\Vert {{\bf{W}}}_{l}\Vert }^{2}+O({\omega }^{-4}\mathrm{)}.$$

Note that the low-frequency limit is fully determined by the connectivity between agents **W**, while the high-frequency limit only depends on the connectivity of the agents with the leader **W**_*l*_. For networks with a fixed outdegree *k*, the norm of this connectivity decreases as ${\Vert {{\bf{W}}}_{l}\Vert }^{2} \sim \mathrm{1/}k$. Thus, at high frequencies, distributed consensus systems have a decrease in responsiveness with increasing number of connections. This is a general behavior and not a particular feature of the regular ring network used in the previous calculations.

In general, there is no such direct relation between **W** ^−2^ and the amount of connections in the network, which means that the behavior of the system at low frequencies is more sensitive to the features of the interaction network beyond its outdegree distribution.

From the standpoint of designing artificial swarms, this analysis highlights that the pace of typical perturbations faced by the system is central in defining appropriate levels of interagent connectivity. When subjected to slow-changing perturbations, the system’s effectiveness always benefits from a higher level of connectivity. Comparing with earlier observations, one can deduce that in the low-frequency regime, the system does not require high correlation strengths for good propagation of the signal, but it does benefit from an increase in speed that higher correlation lengths provide. On the other hand, fast perturbations inevitably reduce the system’s effectiveness with increasing interagent connectivity. Extending the comparison, this suggests that in the high-frequency regime, a high correlation strength is paramount for the signal to be effectively transmitted throughout the entire system.

## Discussion

A myriad of organisms manifest swarming and social organization to some degree. It is well known that such collective behaviors notably improve the effectiveness of fundamental tasks, e.g. predator avoidance, foraging, or mating. However, our phenomenological and analytical study of different models of collective behavior reveals that an excess in social interaction can have detrimental effects, in that it leads to a reduced capacity of response to fast, localized perturbations.

Specifically, we have shown that for a system of self-propelled agents–subjected to a consensus protocol to align their velocities–the capacity for collective predator avoidance is maximized when the number of connections is limited to the value corresponding to the peak in correlations. Beyond the field of swarming, we have found that in simulations of a minimalist model of collective decision-making–the linear threshold model–the speed at which the effects of a perturbation spread through the system is reduced if the outdegree is increased above a certain value. Lastly, a frequency-domain analysis within the LTI framework reveals the underlying cause of this phenomenology: in general, adding more connections in a multi-agent system increases its responsiveness to slow perturbations while decreasing its responsiveness to fast ones.

Simulations of an idealized predator attack upon a swarm of SPPs following the Vicsek model with both metric and topological interactions reveal a direct correspondence between the amount of correlations in the swarm and the survivability of its members in hostile environments. This correspondence is reminiscent of the fluctuation-dissipation theorem for equilibrium statistical physics, that relates the integrated correlation of the system to the real susceptibility.

Since in this model the integrated correlation peaks at a given number of connections, the link with predator avoidance observed here shows that tuning the number of connections allows the group to increase its responsiveness and enhance its performance in biologically-relevant functions while maintaining a high degree of alignment. We speculate that this may be the reason behind the apparently self-imposed limit on social activity observed in flocking birds^21^, social ants^22^, and other taxa (see Supplementary Note VII).

In terms of correlations, an increase in the number of neighbors yields an increase in the correlation length at the cost of decreasing the correlation strength. At low number of connections (e.g. below *k* = 20 in Fig. 1(b)) this is a beneficial trade-off for the swarm: the increase in correlation length effectively allows the information to propagate faster through the interaction network. Thus, more agents are capable of responding to the presence of the threat. However, at higher numbers (e.g. beyond *k* = 20 in Fig. 1(b)), the increase in correlation length only affects agents far away from any danger and marginally benefits the overall performance of the swarm. On the other hand, this increase in the correlation length is accompanied by a drastic reduction in correlation strength that, in turn, severely reduces the responsiveness of agents in the vicinity of the threat.

An alternative approach to understand the effect of the level of interagent interaction on collective response is to perform a network-theoretic analysis of the system of SPPs. As the agents move and align their heading with that of their neighbors, they form a dynamic network of interactions^17^. These interaction networks are temporal, homogeneous, small-world networks^17^ with a topology–degree distribution, shortest path, clustering coefficient, etc.–strongly dependent on the number of interacting neighbors *k*, which in turn influences the group’s dynamics^18^. The spreading of the fleeing behavior through the SPP interaction network in the presence of a predator parallels the complex contagion processes studied in social networks where, as mentioned, the topology of the underlying network profoundly influences the effectiveness of contagion. For this reason, studying simplified models of decision-making and distributed consensus over different static networks provides valuable insights into how the responsiveness of collective motion is affected by the properties of the interaction.

The number of neighbors has similar effects on responsiveness for the case of multi-agent systems performing distributed consensus with a threshold-triggered dynamics, meaning that an agent only changes its state when a certain ratio of its neighbors do. These kind of threshold events have been observed in the spread of behavior over social networks^19, 27, 28^ and the so-called flash expansion of whirligig beetles facing a potential predator threat^46^.

It is worth pointing out that our results for dynamical responsiveness complement previous studies associated with global properties such as the robustness of the interaction network^16^ or the consensus speed^18^. In these studies, increasing the amount of interaction eventually yields diminishing returns–i.e. less gain per neighbor–but never an actual reduction in the property of interest. Diminishing returns can only justify the preference for a finite number of connections if the cost for establishing links between agents is significant. However, quantifying such costs in biological swarms is close to impossible given the complexity associated with sensory and neurological requirements^16, 18^. In contrast, the present study on the dynamical responsiveness of the swarm shows an absolute reduction in swarming effectiveness when the number of neighbors is increased above a certain level.

This fact raises the important question of why collectives with excessive connectivity display a reduced effectiveness under some scenarios such as a predator attack, but not under others such as consensus reaching. The present analysis of the responsiveness of multi-agent systems following LTI consensus dynamics under time-varying perturbations reveals that one key element for predicting the effect of connectivity on responsiveness is the speed of perturbation changes.

In many cases, being able to react efficiently to perturbations in the appropriate time scale is essential for the performance of systems conducting distributed consensus. For example, ants performing collective transport of food rely on transiently informed peers to locate their nest^32^. These informed “leaders” forget their knowledge after a time of joining the collective action, and thus provide a changing signal with a certain characteristic time scale to the swarm. Successful transport depends both on a high consensus over the direction of movement and a proper responsiveness to this dynamic input.

As can be seen in Fig. 4, high levels of connectivity provide marginal benefits when the system is subjected to slow perturbations, but yield a sizable reduction in effectiveness in the presence of relatively fast perturbations.

In summary, previous studies in the animal realm^8, 21, 22^ and in social systems^19^ provide evidence suggesting that, in some cases, it is optimal for collectives to limit the amount of interaction. We have presented simulations of predator avoidance under the SPP model for collective motion, a numerical study of decision-making dynamics, and an analysis of the frequency-response in a consensus protocol that consistently exhibit a decreased responsiveness associated with an excess of connections or interaction. Given that these models are relatively general and unadorned, we suggest that this non-trivial relation between responsiveness and connectivity may be a general feature of a wide range of complex systems involving distributed consensus.

Besides shedding a new light on our understanding of collective behavior, this has also clear implications for the design of networked systems. Even ignoring the possible costs of establishing connections and transmitting information between agents, it may be desirable to limit the number of connections in order to achieve a more effective dynamical response.

## Methods

### Self-propelled particles

We use the self-propelled particles (SPP) model developed by Vicsek *et al*.^23^ as a minimalist model of collective motion that captures the cooperative alignment of orientation. There are several extensions and improvements to this model that generate more realistic and specific dynamics^47^, but we use the original model for the sake of generality and simplicity. Each particle moves in a two-dimensional periodic space and changes its direction of motion at discrete timesteps in order to align to its neighbors’ mean orientation according to

4 $$\begin{array}{rcl}{{\bf{x}}}_{i}(t+{\rm{\Delta }}t) & = & {{\bf{x}}}_{i}(t)+{\rm{\Delta }}t\,{{\bf{v}}}_{i}(t),\\ {\theta }_{i}(t+{\rm{\Delta }}t) & = & {\rm{\arg }}(\sum _{j \sim i}{{\bf{v}}}_{j}(t))+2\pi {\eta }_{i}(t),\end{array}$$

where the velocity vector ${{\bf{v}}}_{i}={v}_{0}{\hat{{\boldsymbol{\theta }}}}_{i}$ has constant magnitude *v*_0_ and direction *θ*_*i*_, arg() gives the orientation of a vector, and *η*_*i*_(*t*) is a random number uniformly distributed in the [−*η*/2,*η*/2] range. The sum *j* ~ *i* is performed over the neighbors of *i* (including *i* itself).

While the original Vicsek model considers that a pair of agents interact–i.e., are neighbors–if they are closer than a certain distance (metric interaction), there is strong evidence that certain natural systems such as flocks of birds interact with a fixed number of neighbors instead (topological or metric-free interaction)^21, 48^. For this reason, we have studied different kinds of interactions only to find the same phenomenology; the responsiveness depends essentially on the amount of interaction in the swarm, not the details of the interaction rule itself.

The results presented in this work have been obtained by computing the dynamics of a set of *N* = 2,048 SPPs following the Vicsek model starting from random positions and velocity orientations. The numerical calculations have been performed using the libspp library^49^. Further implementation details can be found in Supplementary Note III.

### Correlations

The dimensionless velocity fluctuation is defined as

5 $$\delta {{\boldsymbol{\phi }}}_{i}=\frac{{{\bf{v}}}_{i}-\langle {\bf{v}}\rangle }{\sqrt{\sum _{k=1}^{N}{\Vert {{\bf{v}}}_{k}-\langle {\bf{v}}\rangle \Vert }^{2}/N}},$$

where $\langle {\bf{v}}\rangle ={\sum }_{i=1}^{N}{{\bf{v}}}_{i}/N$ is the average velocity. The connected correlation function is then given by

6 $$C(r)=\frac{{\sum }_{i\ne j}\delta {{\boldsymbol{\phi }}}_{i}\cdot \delta {{\boldsymbol{\phi }}}_{j}\delta (r-{r}_{ij})}{{\sum }_{i\ne j}\delta (r-{r}_{ij})},$$

where ${r}_{ij}=\Vert {{\bf{r}}}_{i}-{{\bf{r}}}_{j}\Vert$ is the distance between agents *i* and *j*, and *δ*(*r*) the Dirac delta distribution. For finite-size systems, one can use the maximum of the cumulative correlation as an estimation of the total correlation in the system^7^,

7 $$\chi \equiv \mathop{{\rm{\max }}}\limits_{{r}_{0}}({\int }_{r < {r}_{0}}C(r)d{\bf{r}}).$$

This integrated correlation is usually referred to as susceptibility. While in collective animal behavior one cannot formally relate this integrated correlation to the response of the system, several studies^7, 50^ have shown a phenomenological relation between *χ* defined as Eq. (7) and the way the group responds collectively to a perturbation.

To obtain numerical values of the correlation function *C* and *χ*, we compute the histogram of the correlations in the system every 5 × 10^3^ iterations during 2 × 10^6^ iterations, after discarding the first 5 × 10^4^ iterations as transient dynamics. The correlation *C*(*r*) shown in Fig. 1(a) is the average over 400 histograms obtained with this procedure.

### Predator attack

The predator is introduced as an agent that does not participate in the consensus protocol. Instead, it is afforded predatory capabilities: it moves 40% faster than swarming agents, systematically in the direction pointing to the closest one. When the predator “catches” an agent, the latter is removed from the simulation. An agent can only detect the presence of the threat when it is located at a distance smaller than a fixed “danger-detection” radius *R*_*D*_; as soon as the agent detects it, an evasive maneuver is initiated with the agent moving away in the direction opposite to the predator. We have set *R*_*D*_ to be constant throughout the simulations and independent of the number of neighbors. The fleeing behavior takes precedence over the collective motion of a particular agent for as long as the predator lies inside its danger-detection area. Thus, the agents in this simulation follow the equations of motion (4) with the exception that

8 $${{\bf{v}}}_{i}(t)={v}_{0}\frac{{{\bf{x}}}_{i}(t)-{{\bf{x}}}_{P}(t)}{\Vert {{\bf{x}}}_{i}(t)-{{\bf{x}}}_{P}(t)\Vert }\quad {\rm{if}}\quad \Vert {{\bf{x}}}_{i}(t)-{{\bf{x}}}_{P}(t)\Vert  < {R}_{D},$$

where **x**_*P*_ denotes the predator’s position.

The mean avoidance time shown in Fig. 1(c,d) is obtained by computing the swarm dynamics in the presence of a single predator (introduced after a transient of 2,000 iterations) for 500 different runs of 5,000 iterations each. The reason for computing several runs instead of running the calculation for longer times is that the results depend on the density of agents in the swarm, and the repeated removal of agents by the predator can cause significant changes in the density after long times.

### Collective decision-making

The linear threshold model is a generalization of the simple majority vote model^36^ where the state of each agent or node *i* is determined by a binary variable *s*_*i*_ = {0, 1}. The dynamics of the model dictates that, at a given timestep *t*, *s*_*i*_(*t*) takes the value 0 or 1 according to

9 $${s}_{i}(t+\mathrm{1)}=\{\begin{array}{ll}1 & {\rm{if}}{\langle {s}_{j}(t)\rangle }_{j \sim i} > \theta \\ 0 & \text{otherwise},\end{array}$$

where ${\langle \cdot \rangle }_{j \sim i}$ is the average over all neighbors of *i* and *θ* is a parameter that determines the minimum ratio of neighbors that need to be in the state *s* = 1 for an agent to switch to it.

To study the effects of a perturbation on the collective decision-making process, we consider the following scenario: a given set of *N* = 2,048 networked agents reside in the “ground” state *s*_*i*_ = 0 ∀*i* when, at *t* = 0, an unspecified perturbation induces a small fraction of “informed” agents to abruptly switch to (and remain in) the state *s*_{*j*}_ = 1. This change propagates through the network and causes more agents to switch from state 0 to 1. If the fraction of initially informed agents is large enough and the network is connected, the mean polarization *P*(*t*) = 〈*s*_*i*_(*t*)〉 will eventually reach *P* = 1. One can characterize the responsiveness of the decision-making process by the speed at which this change propagates through the system, measured by the rate of change in polarization,

10 $$c=\frac{dP}{dt}=\frac{1}{N}\frac{d}{dt}\sum _{i=1}^{N}{s}_{i}(t).$$

Further details can be found in Supplementary Notes III–V.

### Distributed consensus in multi-agent systems

Let us consider a group of *N* + 1 identical agents performing a distributed consensus protocol on their scalar state-variable *x*_*i*_(*t*), through a connected and undirected network. The system is characterized by the global state vector **X**(*t*) = {*x*_*i*_(*t*); *i* = 0, …, *N*} and the adjacency matrix of the underlying graph **A** = {*a*_*ij*_; *i*, *j* = 0, …, *N*}, where *a*_*ij*_ = 1 if agent *i* is connected to *j* and 0 otherwise. Given a certain connectivity graph, the state of the system evolves according to

11 $$\begin{array}{rcl}\frac{d{x}_{i}}{dt} & = & \frac{{\omega }_{0}}{{k}_{i}}\sum _{j=0}^{N}{a}_{ij}({x}_{j}(t)-{x}_{i}(t)),\\  & = & \sum _{j=0}^{N}{w}_{ij}{x}_{j}(t),\end{array}$$

where *ω*_0_ is the natural response frequency of our identical agents, and ${k}_{i}={\sum }_{j=0}^{N}{a}_{ij}$ is the degree of agent *i*, i.e. its number of neighbors in the network sense. The quantity *w*_*ij*_ = *ω*_0_(*a*_*ij*_/*k*_*i*_ − *δ*_*ij*_) is introduced for the sake of a compact notation for the governing dynamical equations. As is classical with many swarming systems, these dynamics involve relative output information of neighboring agents^41^.

We model the process of distributed transfer of information by considering a leader-follower consensus dynamics. This is implemented by affording one agent–say agent *i* = 0–with a dynamics not abiding by Eq. (11), but instead following an arbitrary trajectory *x*_0_(*t*) = *u*(*t*). This single control input has a direct effect onto the dynamics of its *k*_0_ neighboring agents, but also indirect effects onto the dynamics of many more agents through the coupled set of dynamical equations (11). In the presence of this single leader, Eq. (11) can be recast as

12 $$\frac{d{x}_{i}}{dt}=\sum _{j=1}^{N}{w}_{ij}{x}_{j}(t)+{w}_{i0}u(t),$$

for *i* = 1, …, *N*.

Despite the static nature of the topology of interaction, this leader-follower consensus model is a good idealization of the process of social information transfer occurring in startled schools of fish or flocks of birds, where one individual has access to privileged information about a potential threat or other kind of external perturbation. This temporary leader triggers a wave of agitation that propagates strikingly fast through the swarm^5, 51^. Such waves of agitation are initiated by extremely rapid changes in the leading agent’s state, which very effectively propagate to all other swarming agents^1^.

Within this leader-follower scheme, one can characterize the responsiveness of the multi-agent system undergoing the distributed consensus process as its capacity to follow fast changes in the leader’s dynamics, *u*(*t*). Specifically, with an input signal oscillating at the frequency *ω*, *u*(*t*) = *u*_0_ *e* ^*iωt*^, the state of all agents at long times becomes proportional to *u*(*t*) with a factor given by the transfer function,

13 $${\bf{H}}(\omega )=\mathop{\mathrm{lim}}\limits_{t\to \infty }\frac{{\bf{X}}(t)}{u(t)}={(i\omega {\bf{I}}-{\bf{W}})}^{-1}{{\bf{W}}}_{l}.$$

where **I** is the identity matrix of dimension *N*, **W** = {*w*_*ij*_} is the *N* × *N* consensus protocol matrix and **W**_*l*_ = {*w*_*i*0_} is the *N*-vector resulting from projecting **W** onto the subspace of the leader. This allows us to define the system’s responsiveness as the norm of this transfer function, or gain, ${H}^{2}={\Vert {\bf{H}}\Vert }^{2}={\sum }_{i}{|{h}_{i}(\omega )|}^{2}$, with |*h*_*i*_(*ω*)| ≤ 1 for all *i* and *ω* ^52^. As is clear from Eq. (13), the gain has a nontrivial dependency on the topology of the agents’ connectivity, including that of the leading agent, through the entries of **W** and **W**_*l*_.

## Electronic supplementary material

Supplementary Information

Supplementary Video

Supplementary Video

Supplementary Video
