# Supplementary material for: Effect of Correlations in Swarms on Collective Response
Source: Sci Rep. 2017 Sep 4;7:10388. doi: 10.1038/s41598-017-09830-w (PMC5583190; doi:10.1038/s41598-017-09830-w)
Supplement: Supplementary file 1 — Supplementary Information [file 41598_2017_9830_MOESM1_ESM.pdf]

# Effect of Correlations in Swarms on Collective Response

David Mateo, Yoke Kong Kuan, and Roland Bouffanais
*Singapore University of Technology and Design, 8 Somapah Road, Singapore 487372*

## I. PREDATOR AVOIDANCE MOVIES

Movies M1 to M3 present examples of the predator avoidance simulations for different $k$. In these simulations, a single predator is introduced in a swarm of $N = 2,048$ SPPs moving about a periodic bidimensional box and topologically interacting with a fixed outdegree $k$. The red circle represents the danger-detection area, i.e. the region of space where agents are able to detect the predator (located at the middle of the circle and moving 1.4 times faster than the agents).

**Movie M1:** Optimal predator avoidance at $k = 16$. While the response of a single agent is limited in range to the red circle, the collective is able to respond to the threat posed by the predator at much larger scales—given optimal levels of interaction. This effective improvement in perceptual range can be seen, for example, in the first seconds of the movie where the agents break their straight herding movement for a radial escape formation and drastically reduce the number of agents in a region surrounding the predator several times larger than the single-agent detection area.

**Movie M2:** Sub-optimal (insufficient interaction) predator avoidance at $k = 8$. When the amount of interaction is not large enough, the response of the swarm is limited to the local surroundings of the threat. Nearby agents are able to temporarily avoid the predator. However, due to the small effective interaction range, their behavior does not trigger a system-wide response that allows for a collective global predator avoidance.

**Movie M3:** Sub-optimal (excessive interaction) predator avoidance at $k = 40$. When the amount of interaction is too high, the swarm's response to the threat is too global and not effective enough in the area surrounding the predator. Note that the swarm is indeed responding at large scales, as evidenced by the global shift in the collective heading. However, due to the long range of the interaction, the information received by the agents surrounding the predator is not local enough for them to perform an efficient predator avoidance.

These results can be reproduced using the example code provided with `libspp` [1] by setting $\Delta t = 1$, $v_0 = 0.04$, $\eta = 0.05$, $\rho = 1$, and specifying the appropriate random seed: 23619 for M1, 28068 for M2, and 7920 for M3.

## II. DISTRIBUTION OF AVOIDANCE TIMES

The mean avoidance time presented in the main text is sensitive to the number of neighbors. By studying the distribution of these avoidance times (Fig. S1) one can see that the improved response of the system is not due to a shift in the peak of the distribution but to the appearance of a heavy tail. In other words, the average performance of the swarm is improved not because the typical time a predator needs to catch a prey is increased but because the optimal number of connections maximizes the probability of rare and large lapses of time during which the predator is unable to catch any prey. Heavy tails, or power-law distributions of rare events, are common features of complex systems. When a system features power-law distributions, it is said to have scale-free cascades: rare but large events that dominate the dynamics of the system. Indeed, in our calculations the heavy tails alone are responsible for the 40 % improvement in the average avoidance time shown in Fig. 3 in the main text.

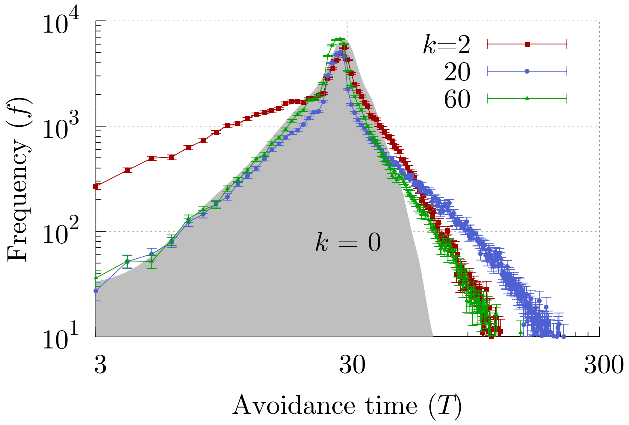


FIG. S1. Avalanche of fleeing agents triggered by the predator. Distribution of avoidance times for a noninteracting swarm (gray filled area) and for topologically-interacting swarms with $k = 2$ (red), 20 (blue) or 60 (green) neighbors. The unnormalized frequency is the total amount of events registered with a given avoidance time. While the distribution peaks at the same value for all $k$, the tail is heavily influenced by the collective behavior of the agents.

These cascades are emergent system-wide responses of the swarm to the threat presented by the predator. While the position of the peak of the distribution depends on the properties of individual agents (namely $v_0$ and $R_D$), the characteristics of the tail depend mostly on the collective properties of the swarm, in particular in the number of interacting neighbors.

In the absence of interaction, the system displays a distribution of avoidance times with an exponential decay (gray shaded curve in Fig. S1). However, a very small amount of interaction—say two neighbors ($k = 2$)—is sufficient for the distribution to drastically change and develop a heavy tail. Quite interestingly, by fitting the tails to a power law (see Fig. S2) we find that the exponent of the tail also peaks at the optimal number of connections.

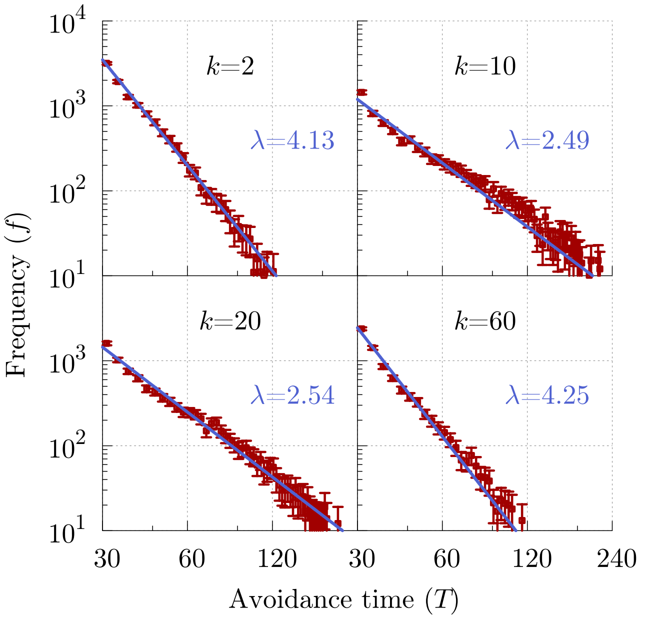


FIG. S2. Power law fitting of the avoidance time distributions, $f = \mu T^{-\lambda}$. Even though the range of times is not enough to determine if the data follows a power law, the exponent $\lambda$ still provides a measure of how fast the frequency $f$ decreases.

## III. IMPLEMENTATION DETAILS

The SPP model for agents following a Vicsek consensus protocol in a two-dimensional space contains several parameters: the number of agents $N$, the speed of the agents $v_0$, the update time $\Delta t$, the swarm density $\rho$ (or, alternatively, the box size $L = \sqrt{N/\rho}$), and the amount of noise $\eta$. We can define the units of length and time through these parameters and thus fix, without loss of generality, $\Delta t = 1$ and $v_0 = 0.04$. We have chosen to set a density of $\rho = 1$. These $\Delta t, v_0$, and $\rho$ are close to what has been traditionally used in the original study of the Vicsek model [2, 3] and they provide reasonable convergence times in the magnitudes considered.

Throughout the work we present results obtained with $N = 2,048$ and $\eta = 0.04$. For large enough $N$, the number of agents does not seem to critically influence the results beyond a scaling factor. The results are not very sensitive to the precise value of the noise level $\eta$ as long as this is low enough to keep the swarm in a highly ordered state. However, lowering $\eta$ to arbitrarily small values is prohibitively taxing from the computational perspective, as it increases the transient and sampling periods needed to obtain statistically representative data.

The correlation function $C(r)$ is obtained by performing an histogram of the quantity $\delta\vec{\varphi}_i \cdot \delta\vec{\varphi}_j$ over 200 bins between $r = 0$ and $r = L/\sqrt{2}$. One can choose to compute $\chi$ either through the mean of these 200 histograms or by averaging over the integrated correlation obtained from each one. Both approaches yield compatible results within the estimated error of each other.

We have computed $\chi$ following the framework presented in Ref. [4]. In there, the authors define the susceptibility as the maximum integral of $Q(r)$, and then assert that it is equivalent to the integral of $C(r)$ (bar a constant, see Supplementary Information of the cited work) assuming no strong density fluctuations. We presented Eq. (7) as the definition for the sake of a compact notation, but performed the numerical computations with the original formula (integral of $Q$) for the sake of accuracy.

### A. Sensitivity to parameters

Figure S3 shows the value of $\chi$ for different values of $N$, $\eta$, and $\rho$. The integrated correlation is proportional to $N$, and its peak shifts slightly with increasing $N$. Similarly, it is only slightly affected by the amount of noise in the system for small radii, while being insensitive to noise at larger radii. It is also mostly insensitive to the global density $\rho$ besides the scaling factor in the nondimensional interaction radius $R\sqrt{\rho}$.

Figure S4 shows the avoidance time in the predator attack for different values of $N$ and $\eta$. Density changes are not considered, as consistency would require to also modify the density of predators; having more than one predator adds additional complexity in the model (e.g. the behavior of agents when detecting several predators, a measure of avoidance time that incorporates simultaneous predators) that is besides the scope of the work and whose value is questionable. The avoidance time does increase slightly but consistently with increasing $N$, as it does with decreasing $\eta$. Notice that the results for $\eta = 0.04$ and 0.02 are compatible with those obtained in the absence of noise.

We have limited this sensitivity analysis to the metric interaction, leaving out the topological because *(i)* the data suggests the results are equivalent for both interactions (see Figs. 3(c) and 3(d)), and *(ii)* the computational cost of using the metric interaction is significantly lower than using the topological one.

Regarding the linear threshold model, the simulations have only two free parameters, the number of agents $N$ and the ratio of informed agents (those initially placed at $s = 1$). Fig. S5 shows that, for large enough systems, the results are independent of $N$. For a discussion on the effect of different number of informed agents, see Sec. IV.

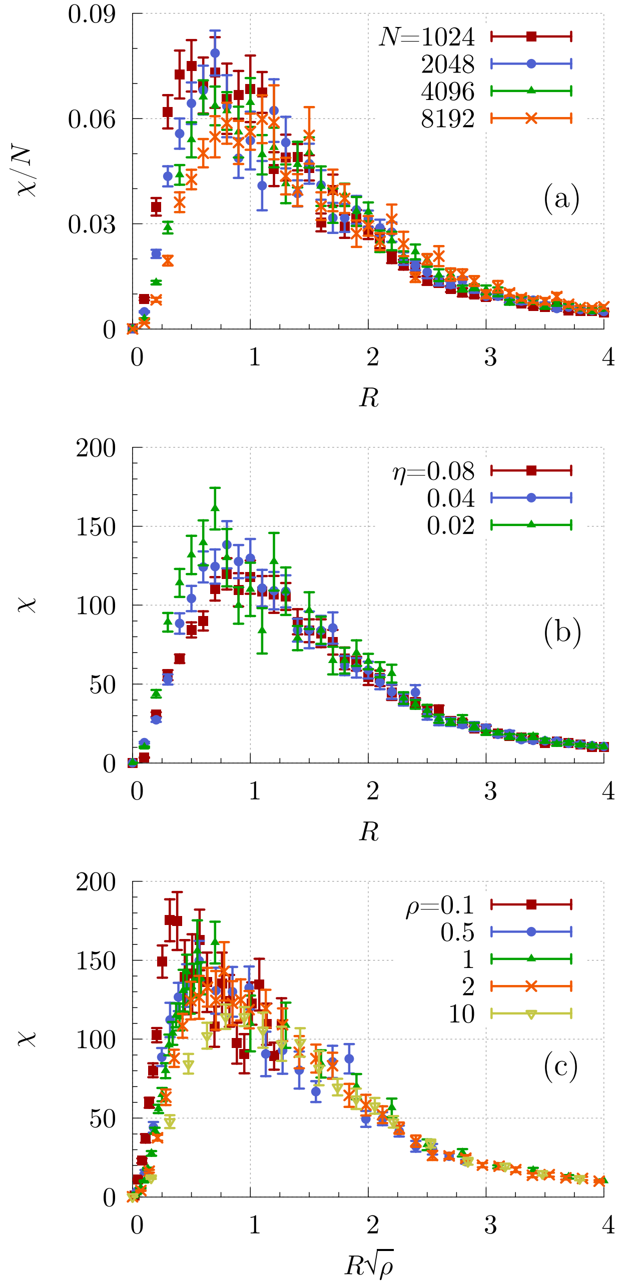


FIG. S3. Sensitivity analysis of the integrated correlation $\chi$ to the free parameters of the model: number of agents $N$, noise $\eta$, and total density $\rho$. (a) Sensitivity to $N$ for fixed $\eta = 0.02$ and $\rho = 1$. (b) Sensitivity to $\eta$ for fixed $\rho = 1$ and $N = 2,048$. (c) Sensitivity to $\rho$ for fixed $N = 2,048$ and $\eta = 0.02$.

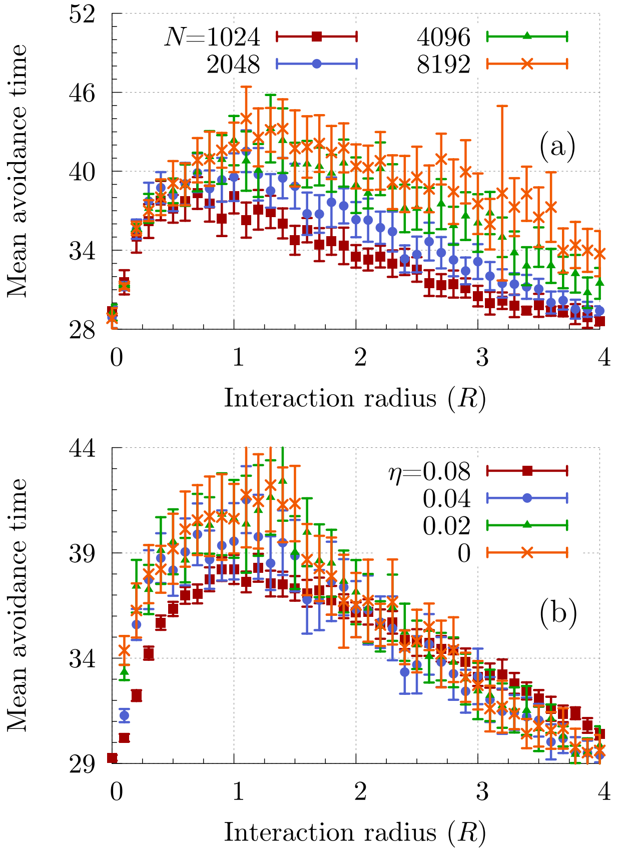


FIG. S4. Sensitivity analysis of the mean avoidance time to the number of agents $N$ and noise $\eta$. (a) Sensitivity to $N$ for fixed $\eta = 0.02$ and $\rho = 1$. (b) Sensitivity to $\eta$ for fixed $N = 2,048$ and $\rho = 1$.

### B. Mean number of neighbors

In the Vicsek model with topological interaction, the number of neighbors is by definition fixed to a certain value $k$, usually labeled as the "outdegree" for its interpretation in the context of graph theory. In the metric interaction with interaction radius $R$, the mean number of neighbors can be roughly estimated by $\langle k \rangle = \rho \pi R^2$.

However, this estimation assumes uniform density and the dynamics of the model will typically take a collective far from uniform density. Figure S6 shows the mean number of neighbors of 2,048 agents following the Vicsek consensus with metric interaction of radius $R$. This data has been used in Fig. 3(c) to compare the mean avoidance time in both interactions.

### C. Avoidance time and correlations

Figure 3(d) of the main text presents the relation between avoidance time and the integrated correlation by including results from a wide range of configurations. These configurations are detailed in Table S1.

For the sake of completeness, the same data is presented again in Fig. S7 using three different classifications: by number of agents $N$, noise $\eta$, or average number of neighbors $\langle k \rangle$.

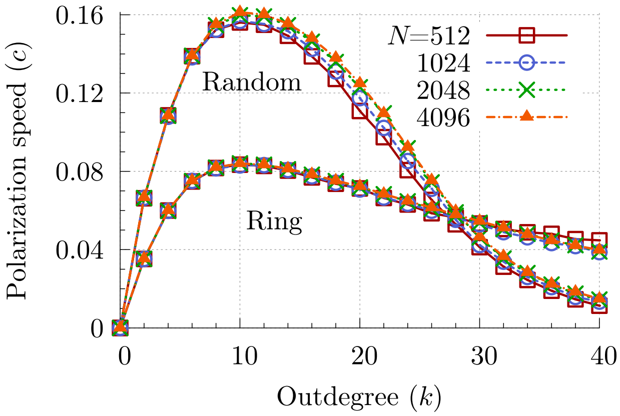


FIG. S5. Sensitivity of the polarization speed to the number of agents $N$.

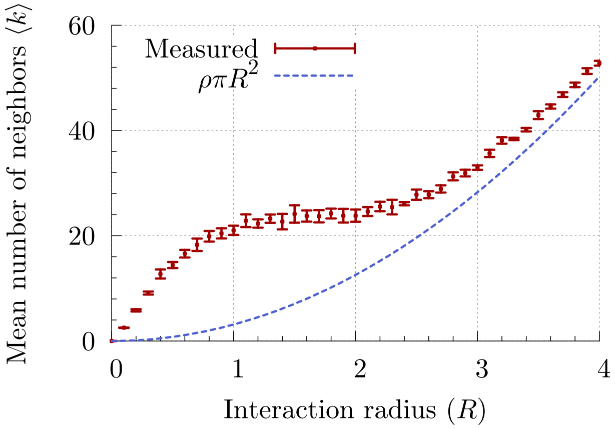


FIG. S6. Mean number of neighbors for a collective of $N = 2,048$ SPPs performing Vicsek consensus with metric interaction, density $\rho = 1$ and noise $\eta = 0.04$. The dashed line corresponds to the uniform density estimation.

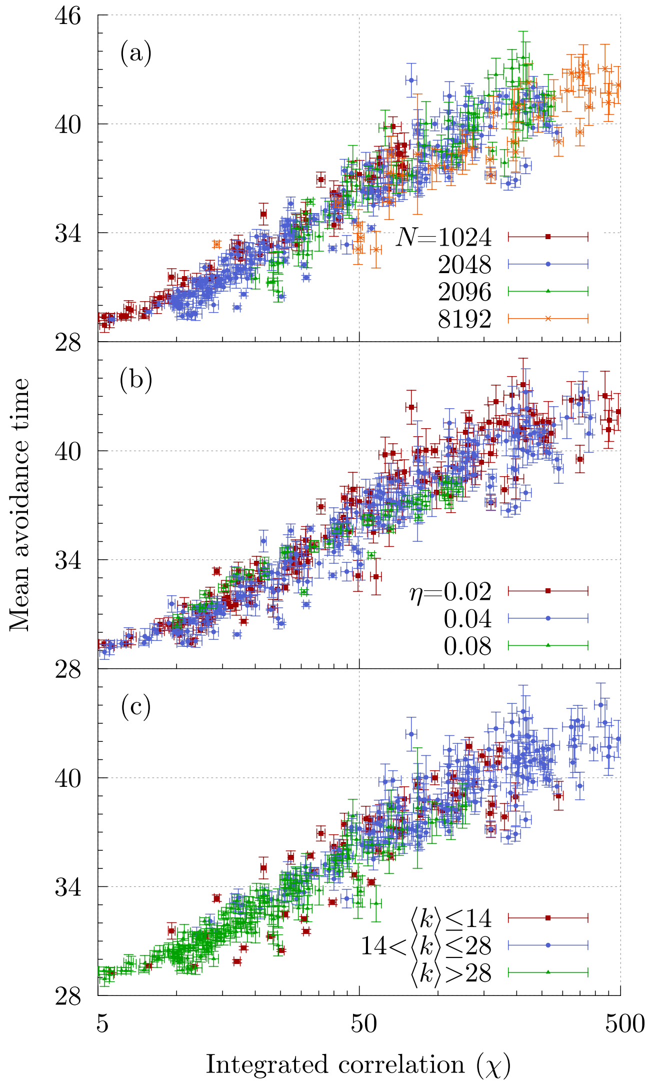


FIG. S7. Relation between mean avoidance time and integrated correlation. The three panels display the same data as Fig. 3(b) in the main text, classified by (a) number of agents $N$, (b) noise $\eta$, and (c) mean number of neighbors $\langle k \rangle$ (exact outdegree for the case of topological interaction).

## IV. FRACTION OF INFORMED AGENTS

The initial condition of the linear threshold model calculations is that a certain amount of agents have access to privileged information so that they start at $s = 1$ instead of $s = 0$. The polarization speed is a measure of how the rest of agents respond to these informed agents or "leaders". Figure S8 shows how this speed is affected by the ratio of informed agents.

Interestingly, the amount of leaders influences the polarization speed profile in the same way that the perturbation frequency influences the LTI response—Fig. 7 in the main text. If the amount of informed agents is low (below 20%), the polarization speed monotonically decreases with increasing connectivity. If it is high (above 45%), the speed increases with connectivity instead. For the intermediate range, the system features a finite optimal connectivity as discussed in the main text.

| Interaction | $N$ | $\eta$ |
|---|---|---|
| Metric | 1024 | {0.02, 0.04} |
| Metric | 2048 | {0.02, 0.04, 0.08} |
| Metric | 4096 | {0.02, 0.04} |
| Metric | 8192 | {0.02, 0.04} |
| Topological | 2048 | {0.02, 0.04} |

TABLE S1. List of configurations used in the relation between avoidance time and $\chi$ (Fig. 3(d) in the main text). For the metric interaction we have considered $R \in [0.10, 4]$; for the topological, $k \in [1, 60]$.

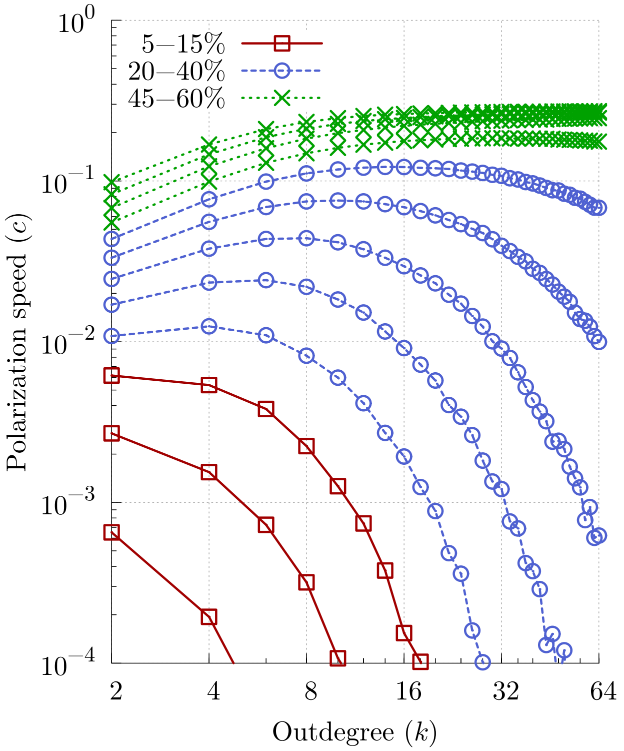


FIG. S8. Polarization speed as a function of the outdegree for different ratios of informed agents. The lowest line correspond to a 5% ratio and each other corresponds to a 5% increase with respect to the one below it. Each speed profile falls in one of three possible phenomenologies: either monotonically decreasing with $k$ (solid red lines), having a peak at a finite $k$ value (dashed blue lines), or monotonically increasing with $k$ (dotted green lines).

## V. RANDOM NETWORKS

We have shown that the polarization speed in the linear threshold model is reduced when the amount of interaction between agents (measured by the outdegree of the interaction network) is increased above a certain value. We have presented results for two kinds of networks, *(i)* a regular periodic one-dimensional grid (a ring) where each agent is connected to its $k$ nearest neighbors, and *(ii)* a completely random network with fixed outdegree $k$ where each agent is randomly connected to exactly $k$ other agents. While the former is a highly regular, undirected network with a high clustering coefficient and a large shortest connecting path, the latter is a stochastic directed network with low clustering coefficient and a small shortest connecting path. These represent two vastly different models from a network topology standpoint.

There is a wide landscape of network typologies that are not necessarily bookended by the two network models considered. For instance, the Watts–Strogatz model [5] is an algorithm generating small-world networks where one starts from the regular ring mentioned above and randomly reconnects each node with a probability $p$. It has been shown that, for certain $p$ values, the networks can have features that neither the regular ($p = 0$) nor the completely random ($p = 1$) networks display, such as having simultaneously a high clustering coefficient and a small shortest connecting path. The original model considers undirected edges, and generates graphs with a nonconstant degree distribution.

We have computed the polarization speed using graphs generated both with the original Watts–Strogatz model and with a modified version that considers directed edges. In this modified version, the end-node of each edge is changed with probability $p$ to a randomly chosen agent (avoiding duplicates and self-links), which allows us to keep a constant outdegree $k$ for all $p$ values.

As can be seen in Fig. S9, the reduction of speed with excessive interaction does not only appears in the regular ring topology ($p = 0$) and the completely random graph ($p = 1$), but also for intermediate values of $p$.

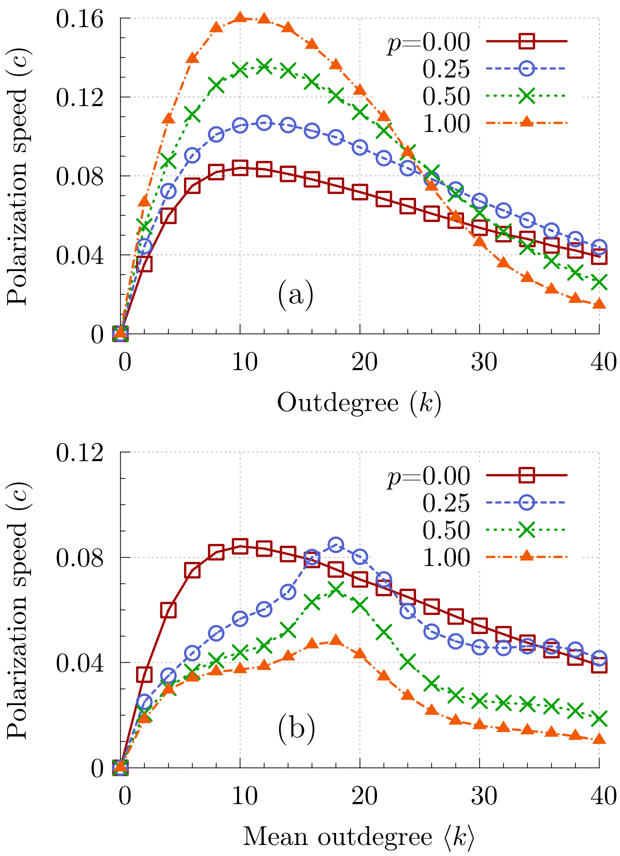


FIG. S9. Polarization speed in the linear threshold model using Watts–Strogatz networks. (a) Modified Watts–Strogatz model with directed edges and fixed outdegree $k$. The networks with $p = 0$ and $p = 1$ correspond to those presented in the main text as "Ring" and "Random", respectively. (b) Original Watts–Strogatz model with undirected edges. In this case, the mean outdegree $\langle k \rangle$ is the outdegree of the agents in the original regular network, before rewiring.

Interestingly, for the same value of $p$, the speed is typ-

ically lower for undirected networks (Fig. S9(a)) than for directed ones (Fig. S9(b)). The optimal outdegree (the peak in speed) is slightly higher in the former case. However, one should be careful in comparing the outdegree in these two models, as $k$ in the directed case is the exact outdegree of every agent while in the undirected case it is only an average quantity.

## VI. LOW AND HIGH FREQUENCY LIMITS

In the main text, we have presented an analytical expression for the gain of an LTI distributed consensus in the limit of low and high frequencies. For the sake of compactness, the expressions in the main text assume the network is undirected. Relaxing this assumption, one gets that the low-frequency limit is

$$\begin{aligned}\lim_{\omega\to 0} H^2 &= |(i\omega - W)^{-1}W_0|^2 \simeq |W^{-1}\left(1 + i\omega W^{-1} - \omega^2 W^{-2} + \ldots\right) W_l|^2 \\ &= |W^{-1}W_l - \omega^2 W^{-3}W_l|^2 + \omega^2 |W^{-2}W_l|^2 \\ &\simeq |W^{-1}W_l|^2 - 2\omega^2 (W^{-1}W_l)^\dagger (W^{-3}W_l) + \omega^2 |W^{-2}W_l|^2 \\ &= |H_0|^2 - 2\omega^2 H_0^\dagger W^{-2} H_0 + \omega^2 |W^{-1}H_0|^2 \\ &= |H_0|^2 + \omega^2 H_0^\dagger \left(-2W^{-2} + W^{-1\dagger}W^{-1}\right) H_0 + O(\omega^4), \end{aligned} \tag{S1}$$

where $H_0 = W^{-1}W_l$ and $|X|^2 = X^\dagger X$. For symmetric systems with $W^\dagger = W$ we recover the expression in the main text,

$$\lim_{\omega\to 0} H^2 = |H_0|^2 - \omega^2 H_0^\dagger W^{-2} H_0 + O(\omega^4). \tag{S2}$$

In the high-frequency limit, the gain only depends on $W_l$, as can be seen from

$$\begin{aligned}\lim_{\omega\to \infty} H^2 &= |(i\omega - W)^{-1}W_l|^2 = \omega^{-2}|\left(1 + i\omega^{-1}W\right)^{-1}W_l|^2 \\ &\simeq \omega^{-2}|\left(1 - i\omega^{-1}W - \omega^{-2}W^2 + \ldots\right) W_l|^2 \\ &= \omega^{-2}|W_l|^2 + O(\omega^{-4}). \end{aligned} \tag{S3}$$

## VII. EVOLUTIONARY DYNAMICS

The predator attack simulations show that limiting the amount of interaction can increase the effectiveness of collective predator avoidance. If agents are able to avoid an attack for longer times by having a particular number of neighbors that maximizes $\chi$, it is reasonable to expect that natural selection would tune the behavior of animals so that they interact with a fixed number of neighbors, as starlings seem to. However, evolutionary pressure selects strategies optimal for the individual and not necessarily the group.

It has been argued [6] that this process of individual optimization in contrast with group optimization can eventually result in collective systems evolving towards total unresponsiveness. The predator avoidance time presented in the main text is a group-level measure of efficiency, and therefore it is not necessarily a predictive indicator of the number of connections that natural systems may develop when submitted to the selective pressure of predators.

We have performed evolutionary dynamics simulations using the same SPP model to asses if evolutionary pressure tunes the amount of interaction to a finite value. At each run, every SPP is initialized with a random position and a random interaction radius. After a transient of $10^4$ iterations, a predator is added and the collective evolves according to the equations presented in the main text. Every time an agent is captured by the predator, the former is removed from the calculation and a new agent is added; the new agent is placed at the antipodal position of the predator, with an interaction radius randomly chosen among those of the SPPs. This means that the probability of new agents having a given radius is proportional to the current number of agents in the swarm with said radius. In order to minimize the effects

of the initial configuration and to have a vast exploration of the state space, new agents have a 0.1 probability of mutating their interaction radius by ±0.025 when they are added to the swarm.

Fig. S10 shows the long-time distribution of interaction radii among a population of 2,048 SPPs with one predator agent. The distribution is obtained by sampling after $4.8 \times 10^6$ iterations and averaging over 50 independent runs. The duration of the simulation guarantees that the number of agents replaced (typically on the order of $10^5$) is much larger than the number of agents in the swarm.

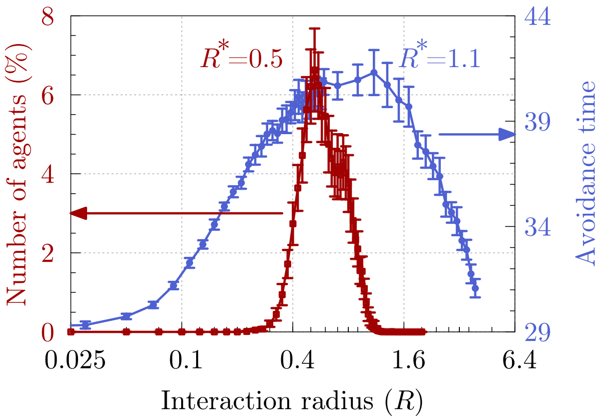


FIG. S10. Interaction radius distribution of an evolving population facing a predator after $4.8 \times 10^6$ iterations. The optimal interaction radius for the individual, achieved through selective pressure, is lower than the collective optimal one, measured by the group avoidance time.

Despite the large dispersion in the data, the simulation consistently yields swarms where 95% of the agents have interaction radii within $0.30 < R < 1.05$, with a median of $R^* = 0.5$ that corresponds to about $\bar{k} = 14$ neighbors per agent. This evolved $\bar{k}$ is significantly lower the one that maximizes the group's avoidance time ($R = 1.1$, or about $\bar{k} = 20$ neighbors).

Interestingly, this basic model of evolutionary dynamics features selective pressure tuning the number of neighbors in the swarm to a finite value, even though this evolved number is lower than the group-optimal one measured by the predator avoidance time.

## VIII. SOURCE CODE

The numerical calculations of SPP dynamics have been obtained using the `libspp` library. The source code of the library, along with some example usages to compute the correlations and the predator simulations, is available at [1]. The latest version of the library is available at `https://github.com/david-mateo/swarming-spp/`.

All calculations related to the linear threshold model decision-making and the LTI distributed consensus protocol, including the generation of adjacency matrices representing the different types of networks, have been performed with a collection of Octave [7] functions available at `https://github.com/david-mateo/multiagent-consensus/`.


[1] Mateo, D. swarming-spp: A library for simulations of self-propelled particles and the theoretical study of emergent swarming behaviors. (2015). URL `http://dx.doi.org/10.5281/zenodo.30587`.

[2] Vicsek, T., Czirók, A., Ben-Jacob, E., Cohen, I. & Shochet, O. Novel type of phase transition in a system of self-driven particles. *Phys. Rev. Lett.* **75**, 1226–1229 (1995).

[3] Vicsek, T. & Zafeiris, A. Collective motion. *Physics Reports* **517**, 71 – 140 (2012). Collective motion.

[4] Attanasi, A. *et al.* Collective behaviour without collective order in wild swarms of midges. *PLoS Comput Biol* **10**, e1003697 (2014).

[5] Watts, D. J. & Strogatz, S. H. Collective dynamics of 'small-world' networks. *Nature* **393**, 440 (1998).

[6] Torney, C. J., Lorenzi, T., Couzin, I. D. & Levin, S. A. Social information use and the evolution of unresponsiveness in collective systems. *Journal of The Royal Society Interface* **12** (2014).

[7] Eaton, J. W., Bateman, D. & Hauberg, S. *GNU Octave version 3.0.1 manual: a high-level interactive language for numerical computations* (CreateSpace Independent Publishing Platform, 2009). URL `http://www.gnu.org/software/octave/doc/interpreter`. ISBN 1441413006.
